# ‘Closing the loop’: re-audit of the diagnosis and management of vitamin B12 deficiency in general practice

**DOI:** 10.1007/s11845-022-03103-w

**Published:** 2022-07-23

**Authors:** O. O’Donnell, J. MacCarthy

**Affiliations:** University Hospital Limerick, Limerick, Ireland

**Keywords:** Closed loop audit, General practice, Ireland, Primary care, Vitamin B12 deficiency

## Abstract

**Introduction:**

Vitamin B12 deficiency is common in Ireland, The Irish Longitudinal Study on Aging (TILDA) survey indicates 12% of over 50s in Ireland are low or deficient. The condition is commonly managed exclusively in general practice.

**Aim:**

The intention of this audit was to establish whether B12 deficiency is diagnosed correctly and whether there was over-treatment of patients.

**Methods:**

The audit was conducted in an urban general practice in midwest Ireland. The primary limitation was the low number of patients. Thirty-five patients were included after practice database searches. An initial audit was performed which compared with the standard, Royal University of Bath: ‘Guidelines for the Investigation & Management of B12 deficiency’.

**Results:**

The recommendations from this audit were to complete follow-on investigations and to switch over patients from IM to oral replacement. Twenty-one patients were then recalled, and investigations were performed. Ten patients were then switched from IM replacement to oral therapy. A re-audit was then completed. The re-audit showed marked improvement in compliance, from 17% (*n* = 6) to 83% (*n* = 29). The reduction in patients on IM therapy will decrease practice burden, with an annual reduction of nurse consultations by 46, representing a 30% decrease in nurse consultations for IM vitamin B12. This equates to an annual cost reduction of €1,340.

**Conclusion:**

This closed loop audit demonstrated that there was over treatment and under investigation of patients with B12 deficiency in general practice and that auditing of this process could both reduce risk for patients and save money and time.

## Background

Vitamin B12 deficiency is very common in Ireland, especially among the elderly. The Irish Longitudinal Study on Aging (TILDA), a study of older Irish adults (*n* = 5290), estimated that 12% of over 50s in Ireland are low or deficient in vitamin B12, based on lab values of < 185 pmol/L [1].

As intramuscular (IM) vitamin B12 replacement therapy is the indicated replacement therapy for many patients [2], it can represent a considerable burden on general practice staff, especially the practice nurse. In our urban practice in Midwest Ireland, total patient count approximately 3,000, vitamin B12 replacement represented 4.56% (*n* = 3) of nurse consultations per week, based on 65 nurse consultations per week. This audit was undertaken to establish whether there was over treatment of patients with vitamin B12 deficiency within the practice. The intention of the audit was to identify and change management plans where applicable.

Vitamin B12 is a water-soluble vitamin that is taken in the diet predominantly through animal products, e.g. meat, fish, eggs, and milk. The majority of vitamin B12 is then absorbed via the terminal ileum after forming a complex with intrinsic factor, a glycoprotein produced by the gastric parietal cells. Vitamin B12 deficiency has 3 primary aetiologies [3]:

- Autoimmune: Antibodies targeting gastric parietal cells (anti-parietal cell antibodies) or intrinsic factor (anti-intrinsic factor antibodies) prevent vitamin B12/intrinsic factor complex formation and subsequent absorption of vitamin B12 [3].
- Malabsorption: Inflammation from coeliac disease, terminal ileal resection for Crohn disease, or gastric bypass surgery can result in inability of the intestine to absorb sufficient vitamin B12 [3].
- Dietary insufficiency: Despite large hepatic stores of vitamin B12, deficiency can occur in patients following a strict vegan diet for greater than 3 years [3].

The purpose of this audit was to initially identify whether vitamin B12 is correctly diagnosed within the practice and whether the correct initial management was prescribed. This initial assessment was completed by end of January 2019.

Between the period of January 2019 and June 2019 patients were recalled, further testing was conducted, and management plans were changed accordingly.

In July 2019, data was collected again to assess the interventions made over the previous 6 months. As part of this re-audit, estimates were made of the resource savings after changing over patients from IM replacement to oral replacement.

An audit report was then compiled in order to allow for future comparisons within the practice and also comparison with other practices.

## Methods

### Search criteria

Patients to be included in the audit were identified through searches performed on the practice management software system (Health One©). Keywords searched included ‘vitamin B12 deficiency’, ‘terminal ileal resection’, ‘pernicious anaemia’, ‘hydroxycobalamin’, ‘cyanocobalamin’, and ‘NeoCytamen©’.

A search of vials of hydroxycobalamin and cyanocobalamin stored for patients in the practice was also performed.

### Inclusion criteria

Initial searches revealed a cohort of 41 patients. Each patient was then given a unique identifying number. Subsequently, 6 patients were then removed from the study as 2 had passed away and another 4 had moved on from the practice. This left a total of 35 patients to be included in the audit.

**Fig. 1:**
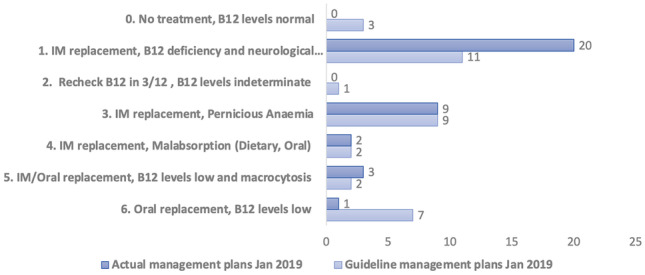
Jan 2019 audit: actual versus guideline management plans

In January 2019, data was collected examining the diagnosis and initial treatment plan (see Appendix 2, Fig. 1 for detail of the data collected for each patient). Once compiled, the data was compared with the standard and compliance was determined using a flag system (see below Table 1 for standard and details).

**Table 1:** Grading of compliance to standard

Full compliance	Partial compliance	Minimal compliance
Diagnosis and initial treatment in accordance with guidelines	Diagnostic criteria met but initial treatment not in accordance with guidelines	Diagnostic criteria not met but treatment offered

### Audit protocol and ethical approval

This audit was carried out as per HSE Healthcare Audit Criteria and Guidance [4, 5] and in-keeping with local University of Limerick guidance. The audit protocol was submitted to the University of Limerick for review prior to commencement of the audit. A request for exemption from ethical approval, based on the grounds of being a clinical audit, was also submitted and approved. At all stages throughout the initial audit, recall, and re-audit, patients were informed and consented to all interventions. As per HSE and local guidance, formal written consent was not obtained from individual patients [4, 5].

The patient information was pseudo-anonymised. The data was analysed using Microsoft Excel, version 16.40.

### Standard and compliance measurement

The following standard for diagnosis and treatment was used for comparison: Royal University of Bath NHS: ‘Guidelines for the Investigation & Management of vitamin B12 deficiency’ [2].

**Fig. 2:**
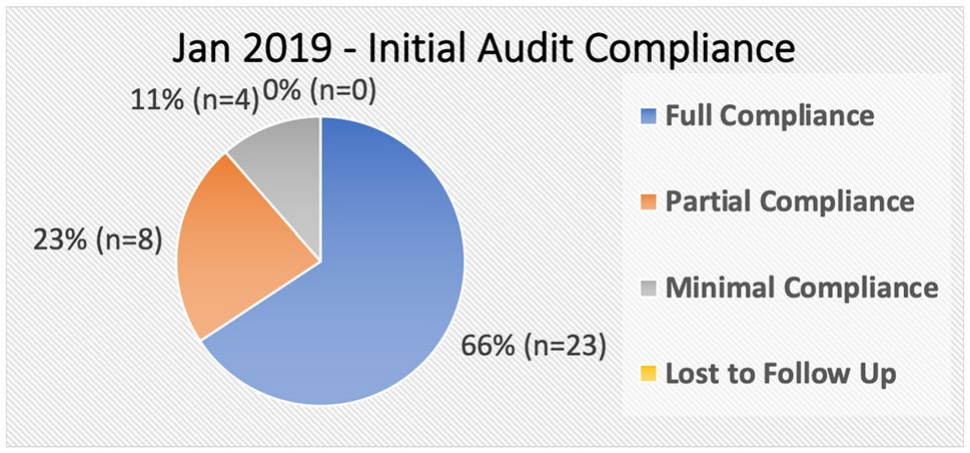
Jan 2019 audit compliance measurement

Note: The lab values given by the aforementioned guidelines have been replaced with the local laboratory reference ranges [6]. The flowchart has been updated accordingly (see Appendix 1, Fig. 2).

The thresholds for compliance were determined as per Table 1.

### Recall criteria

Between January 2019 and June 2019, patients requiring further testing were recalled. The criteria for recall was as follows:

- Last serum B12 result > 12 months previous
- No serum B12 result since initiation of IM hydroxycobalamin injections/oral vitamin B12 supplementation
- No previous serological testing
- Initial Jan 2019 audit recommended change to management plan

Twenty-one patients were recalled for further testing. During these consultations, proposed changes to their management plans were discussed and agreed depending on the results. Once results of the testing were available, patients were contacted, and changes to their existing management plans were confirmed.

### Re-audit

**Fig. 3:**
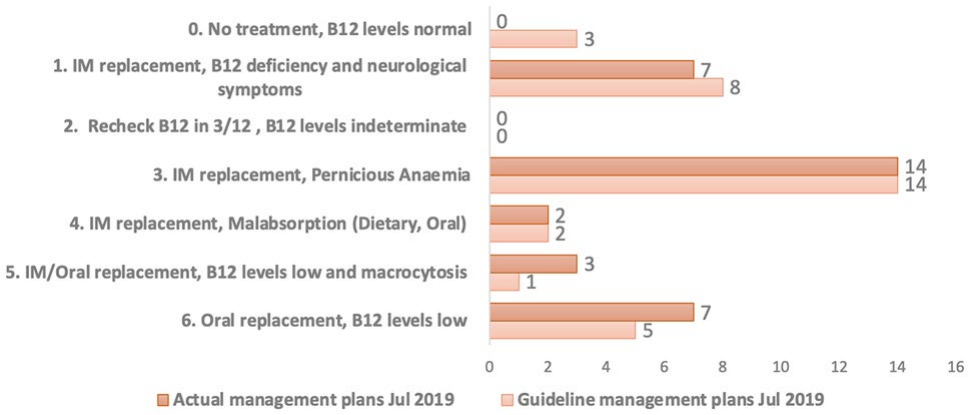
Jul 2019 audit: actual versus guideline management plans

In July 2019, a re-audit was performed. Data was again collected for each of the 35 patients (see Appendix 3, Fig. 3 for data collection form). The results were then compared against the standard and the initial audit data from 6 months previous.

## Results

**Fig. 4:**
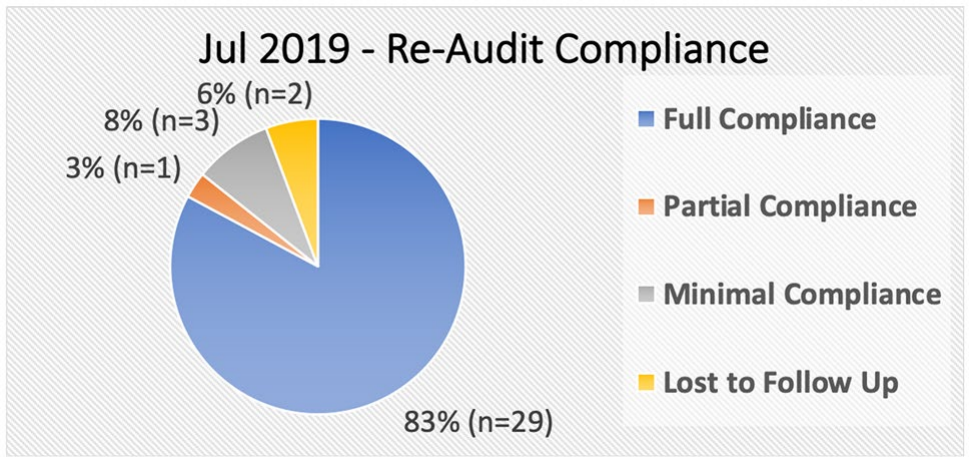
Jul 2019 audit compliance measurement

**Fig. 5:**
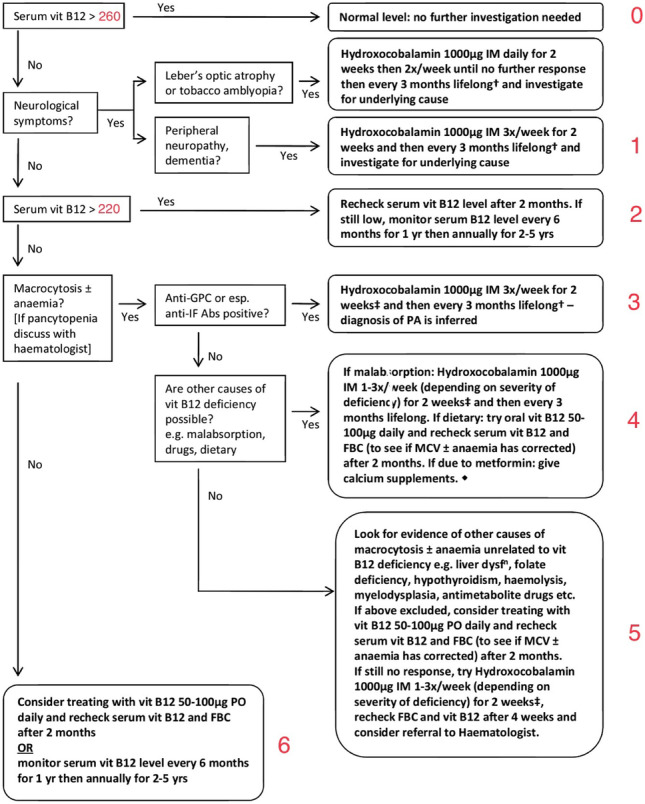
Modified NHS bath flowchart

The results (see Figs. 4 and 5), of the January 2019 initial audit showed that of the 35 patients audited:

- Twenty-three (65.7%) were in full compliance with the standard.
- Eight (22.9%) were in partial compliance with the standard.
- Four (11.4%) were minimally compliant with the standard.
- There were 0 patients who, by the standard, met the diagnostic criteria but were not treated.

Initial assessment of practice burden was based on the regular 3 monthly IM replacement therapy for the patients on that treatment and newly diagnosed patients receiving induction therapy. The following assumption is made to calculate the amount of additional nurse consultations required for the induction treatment. From our data in 2018, 8.5% (*n* = 3) of patients are newly diagnosed per year, this figure was used for cost estimates.

The practice burden for IM replacement therapy is calculated based on nurse consultation time only and does not include any knock-on GP consultations or other investigations based on the initial nurse consultation.

The cost analysis showed that of the 35 patients diagnosed with vitamin B12 deficiency, 34 were receiving IM replacement therapy. This equated 136 nurse consultations for maintenance therapy and 18 consultations for induction therapy, an annual total of 154 nurse consultations per year. This was estimated to cost the practice €4,620 annually, based on €30 per nurse consultation (see Table 2 for details).

**Table 2:** Cost and savings analysis

**Description**	**Jan 2019 audit**	**Jul 2019 re-audit**	**Switched from IM to oral**	**Lost to follow-up**
No. of patients on IM therapy	34	22	10	2
Consultation no. for maintenance	136	88	40	8
Consultation no. for induction	18	12	6	0
Annual consultations	154	100	46	8
Annual cost	€4,620	€3,000	€1,380	€240

**Fig. 6:**
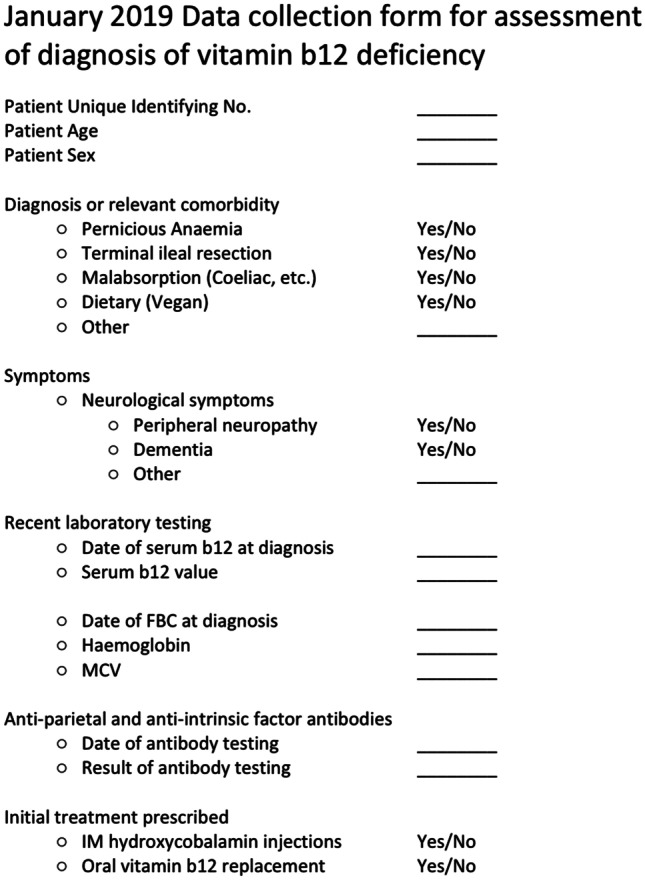
Jan 2019 audit data collection form

**Fig. 7:**
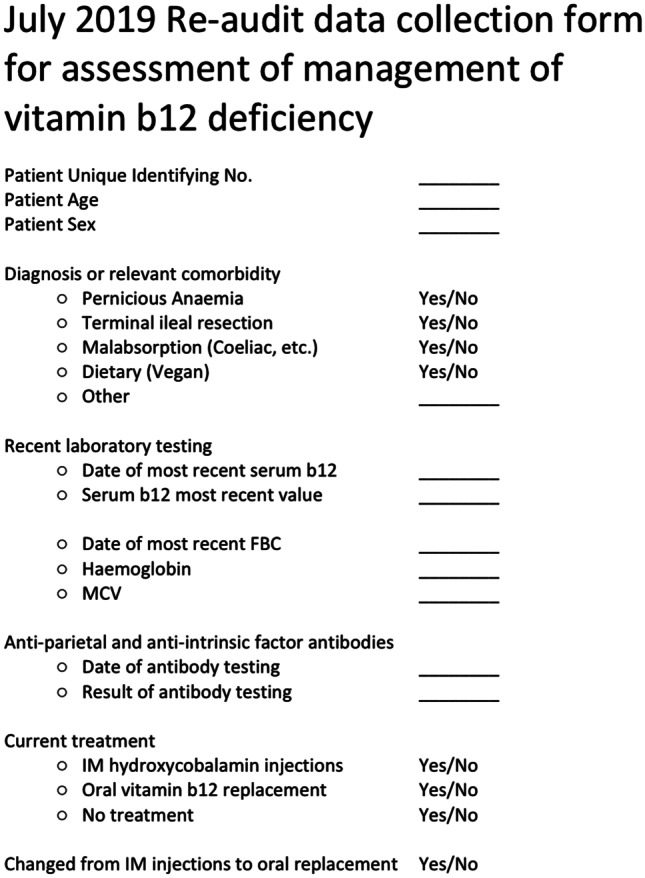
Jul 2019 re-audit data collection form

The results (see Figs. 6 and 7), of the July 2019 re-audit showed that of the 35 patients audited:

- Twenty-nine (83%) were in full compliance with the standard. This represented an increase of 17.3% (*n* = 6).
- One (3%) was in partial compliance with the standard. This represented a decrease of 19.9% (*n* = 7).This patient was being under treated, receiving oral replacement when the standard recommended IM replacement. In this case, the patient has been stable for over 5 years on oral replacement, and it was decided there was no indication to change to IM injections.
- Three (8%) were in minimal compliance with the standard. This represents a decrease of 3.4% (*n* = 1).
  - The 3 patients had serum B12 values greater than 260 and therefore did not meet the criteria for B12 deficiency. Two of the 3 patients suffered from neurological complaints (dementia and falls), and the other had macrocytic anaemia. In all cases, the serum B12 values were only marginally higher than the upper limit of 260, with an average serum B12 value of 287. The 3 patients were moved from IM therapy to oral therapy but remained in the minimal compliance group as they did not meet indications for treatment based on the serum B12 values.
- Two (6%) were lost to follow up. One moved practice, and the other was unable to attend as they work abroad.
- There were 0 patients who, by the standard, met the diagnostic criteria but were not treated.

Of the 21 number of patients recalled, 10 were successfully changed from IM replacement therapy to oral replacement therapy, and another 2 were lost to follow-up. The switchover of patients shall result in an estimated reduction in the annual practice nurse visits by 46, saving a total of €1,380 and reducing patient inconvenience and risk of complications from IM replacement therapy (see Table 2 for details).

## Discussion

The January 2019 audit showed that initial diagnosis was correct in the majority of cases, with 88.6% (31 of 35) being correctly diagnosed with vitamin B12 deficiency. Importantly, there were no patients who met the diagnostic criteria and were not started on treatment.

However, 11.4% (*n* = 4) of patients failed to meet the diagnostic criteria but were commenced on treatment. On closer examination of this cohort, 3 of them have relevant comorbidities (falls, dementia, macrocytic anaemia), and their serum B12 values are only marginally greater than the 260 cut-off point, average 287. At time of diagnosis, the decision was made to start them on IM replacement therapy to address the neurological symptoms/macrocytic anaemia.

While the diagnosis can be deemed as correct for the majority of cases, the January 2019 audit identified many patients who were not started on the correct management plan as per the standard. Thirty-four percent (*n* = 12) of patient’s initial management plans were not in full compliance with the standard. Of the 12 patients, 11 were being over treated, receiving IM replacement where the standard indicated oral replacement. This confirmed the initial assumption prior to the audit that there was over treatment of patients with vitamin B12 deficiency.

One of the most notable findings of the initial January 2019 audit was that there was a large cohort of patients who did not have sufficient follow-on testing. This resulted in 60% (*n* = 21) of patients requiring re-call for further testing in the following 6 months.

With the recall of 21 patients for further testing the subsequent, results allowed for 10 patients who were previously receiving IM replacement to be changed to oral replacement therapy. Interestingly, of the 11 patients identified as being over treated in the January 2019 audit, 6 of these patients were diagnosed with pernicious anaemia after follow-on testing. This further reinforces the importance of conducting follow-on testing after initial diagnosis of vitamin B12 deficiency.

The July 2019 re-audit showed marked improvement in compliance from the initial January 2019 audit. Full compliance with the standard increased by 17% (*n* = 6) to 83% (*n* = 29). The outstanding 17% (*n* = 6), not in full compliance, is comprised of patients lost to follow-up (6%, *n* = 2), and a patient who was deemed to be under treated based on the standard but was clinically stable on oral replacement (3%, *n* = 1). The remaining 8% (*n* = 3) discussed above as having relevant comorbidities of dementia, falls, and macrocytic anaemia were changed from IM replacement to oral replacement.

The re-audit showed that, other than the 2 patients lost to follow-up, all patients were in compliance with the standard or were prescribed alternative treatment based on their individual circumstances.

Analysis of resource saving showed the switchover of 26.8% (*n* = 10) of patients reduced annual nurse consultations by 46. Weekly, this represents a 1.36% (*n* = 1) reduction in nurse consultations, based on a total of 65 consultations per week. The reduction equated to an annual saving of €1,380 to the practice, reduced patient inconvenience, and reduced potential complications associated with IM replacement therapy.

The main limitation of the audit is the low number of patients (*n* = 35). Another limitation is that the applicable standard that used different lab values was from a different region and healthcare system, Royal University Hospital Bath, NHS Trust [2]. Although there are a low number of patients in the audit, we believe this is reflective of the proportion of patients with vitamin B12 deficiency in a rural general practice. As such, we believe that the results of this audit may be generalizable to other similar general practitioners.

Both the initial audit and the re-audit emphasize the importance of a full work-up for vitamin B12 deficiency, ensuring all that follow-on testing is completed.

Another recommendation would be the development of local standards to put a clear framework for diagnosis and management of vitamin B12 deficiency that includes the local laboratory values.

Further direction of the audit may include re-auditing the cohort in 12 months’ time to assess the progress of patients switched over to oral treatment.

Another direction may include screening for B12 deficiency within the practice. This audit identified 41 (1.2%) patients of the 3000 patients in the practice as having vitamin B12 deficiency. The TILDA study [1] indicated that 12% of over 50 s are low or deficiency in vitamin B12. Selective screening of at risk (over 50, malabsorption, bowel resection, long-term vegan) patients in the practice may identify further patients with vitamin B12 deficiency as the current number of patients diagnosed is less than the average as suggested by the TILDA study [1].

A 2018 Cochrane review article [7] suggested that high-dose (1000–2000 µg/day) oral vitamin B12 replacement may be as effective IM vitamin B12 replacement. Depending on further research in the area, a cohort of patients could be selected to trial high-dose oral vitamin B12 replacement instead of IM replacement therapy within the practice.

## Conclusions

The initial concern that prompted the audit was that patients with vitamin B12 deficiency in the practice were being over treated. This assumption was shown to be correct, and efforts to reduce over treatment were successful with 29% (*n* = 10) of people changed from IM replacement to oral replacement therapy. The other outcome from the audit was that patients were under investigated and the aetiology of the B12 deficiency was not fully ascertained.

The audit results demonstrated a reduction in practice nurse consultations by 46 annually, equating to a modest annual saving of €1,380. While the practice burden was only modestly reduced, we believe that this method could be reproduced in other primary care facilities with relative ease and result in similar practice burden and cost reductions.
